# Smallpox and Its Management

**Published:** 1899-04

**Authors:** John W. Kenney

**Affiliations:** Cook’s, New Mexico


For Texas Medical Journal.

### Smallpox and its Management.

BY JOHN W. KENNEY, M. D., COOK'S, NEW MEXICO.

Smallpox being epidemic in various parts of the country at the present time, I thought that perhaps a few remarks on the subject might be interesting, my remarks to be drawn from a personal experience with thirteen cases treated during the autumn of last year.

The disease had been raging with considerable severity during the entire summer, in the districts along the different water ways throughout the Territory. When in the fall a case made its appearance in this mining camp, the inhabitants were not surprised, but truly frightened. Being the surgeon for the several mining companies at this place, I was given full power to act as my judgment dictated toward the disease, the mining companies agreeing to stand by me in any action that I might take in the matter. It was necessary that the disease should be stamped out as soon as it was possible, that there might not be any interruption in the operation of the mines. My first thought was to quarantine each house wherein the disease appeared—no one was allowed to enter or leave the premises. The next step was also in the direction of prophylaxis. Accordingly I visited every house in the camp, and vaccinated some two hundred people—mostly Mexicans, and a majority of these were children, the older Mexicans having had smallpox at some time in the past. At first considerable difficulty was experienced in getting the Mexicans to submit to being vaccinated, but they soon learned that a refusal meant an immediate discharge from the company that employed them, and it was not long before all were anxious to have themselves and their families vaccinated. Their amusement at seeing the white people shun them when they had the disease soon changed to one of fear of having their food supply cut short if they did not interest themselves in stamping out the disease.

The first case in the community was brought there by a Mexican family that had been on a visit to some friends afflicted with the malady. A boy about ten years old contracted the disease. The parents kept it quiet, and at the first opportunity sent the boy over to a little town on the Rio Grande. In about ten days a second case developed, and it was only at this time that the first case was reported, the second case developing in a house near by the first. The people felt indignant that a case of smallpox should have been concealed in their midst till sufficient time had elapsed to spread the infection. A mass meeting of the citizens was held, and it was decided to burn the house wherein the first case developed, if I thought that was the surest way of preventing further infection from that point. They were informed that "fire" would destroy all possibility of infection, and accordingly the next day the house was burned, with all its contents. The burning of the house and furniture was not according to law—the written law—but it had a very salutary effect upon the Mexicans, and the citizens were upheld in their action by the courts, as was proved by several attempts to secure damages by the owners of the burned houses.

The disease appeared rapidly for about three weeks. In every instance the source of infection was different—usually could trace it to the visitation of some fruit peddler from an infected district. The houses at which they spent the night were always the ones to furnish the new cases. Noticing this fact, the camp was quarantined and a guard placed at the mouth of the canon leading into the camp. No one was allowed to enter the place, or to leave it, if they had been exposed to the disease. In less than a month there was not a case in the camp, and there has not been any since.

About a month later I was called over to the Rio Members—a rich fruit farming country about twelve miles distant, in which the disease was holding high carnival and deaths were an every day occurrence. Here I saw four cases of confluent variola in its worst form. In this community the disease was propagated and scattered by public funeral services, all the cases that I attended having contracted the disease in an old church whither they went to pay their respects to a dead friend—one that had died of heart disease. It was in this church, however, that funeral services were daily held over the remains of smallpox patients.

Here I cannot resist the temptation to say that a preacher or priest who will hold a public funeral service over a case of confluent variola is either an ignoramus, an idiot or a fiend incarnate. If the former, he should be put out of harm's way; if a fiend incarnate—if for the sake of the few dollars he receives from his ignorant followers, he holds such services, he should have the devil or his imps removed from his body by the old time method of physical torture.

The diagnosis of smallpox is a simple matter after the eruption appears—till that time it is an impossibility. If you are in the midst of an epidemic, you can make a good guess from the initial symptoms. The high temperature, head and backache, scanty urine, fast pulse and feeling of complete prostration, invariably precede the eruption from three to six days. In this altitude—7000 feet above sea level—the initial symptoms usually lasted five or six days, never less than four, while along the river in the farming country the eruption made its appearance earlier—usually at the beginning of the fourth day. Altitude invariably exerts an influence over the disease. The cases observed by me in the mountains were contracted from those in the valley, some twelve miles distant, and were always of a milder type than those observed in the valley, the difference in the altitude being about two thousand feet. The incubative period was also observed to be longer in the mountains, being never less than fourteen days. When the eruption appears, the initiatory symptoms subside or become less intense, only to return in an aggravated degree when the papules become pustules. From the beginning of the eruption to the termination of desquamation varies from twelve to twenty-four days, depending upon the severity of the disease and the treatment administered.

To make a complete clinical picture, I will report two cases as they were noted in my case book.

Case 1.—Nic. V., a Mexican boy, aged 12 years. Complained of nausea and pain in back and head. The temperature was found to be 104° F. This continued for six days, when the characteristic shot-like eruption made its appearance on forehead and rapidly extended downward over his face, body and extremities. It was of the semi-confluent type. The eruption brought with it temporary respite from the initial symptoms, and passed rapidly through the papular, vesicular, pustular and desquamative stages, desquamation being complete at the end of the seventeenth day of the disease.

Case 2.—John E., an American, well to do and engaged in the fruit growing business. Had attended funeral services in a church twelve days before. Was taken with severe pains in loins and head —nausea and vomiting quickly followed. Temperature rose to 105° F. Urine was almost completely suppressed. Three days later the eruption appeared on forehead and face, and extended over entire body, soon becoming confluent. Restlessness, delirium and convulsions followed in rapid succession. At the end of twenty-four days desquamation had practically been completed. Furuncles or abscesses appeared in the muscles of his arms and legs for about two weeks, when the lower lobe of his right lung became solidified. The inflammation, fortunately, did not extend to the other lobes, and he finally recovered from his long train of Job like afflictions.

The treatment given these patients, and the other eleven treated by me, was almost entirely symptomatic. It was, however, very satisfactory, as they all recovered, while deaths were an every day occurrence on the river, where I saw my four worst cases, the Mexicans over there, as a rule, not desiring any medical attention in such cases. The treatment was as follows: The pain and high temperature in the initial stage was relieved by administering powders composed of acetanilid and caffeine. A saline purgative was given as soon as the nausea would permit. The itching and other disagreeable symptoms of the suppurative and desquamative stages were relieved by the application of a solution of bichloride of mercury to the entire affected areas. Of the bichloride, a 1 to 2000 solution was used. A one to forty carbolic acid solution was also used at times in like manner. After the application of either lotion, the body was dusted with pulverized boracic acid. These applications were repeated twice daily, if necessary. Heart stimulants were given in all cases, from the beginning of the eruptive stage, strychnine and nitroglycerine being the ones used by me. The diet was restricted to liquids, and stimulants entered freely into it.

In those cases where delirium persisted in spite of the antiseptic lotions, I had the patients placed in a hot bath and all the crusts remover that could possibly be detached without causing the patient too much pain. In the few cases that I practiced this, it acted like magic. Pus and crusts should not be allowed to remain on the patient when they can possibly be removed. Strict attention to this, and the free use of antiseptic, will save many a life that would otherwise be lost.

To disinfect the rooms occupied by smallpox patients, I used sulphur and chloride of lime. The ceiling, walls and floor of the rooms were made damp with a solution of the chloride of lime, after which four pounds of sulphur was burned for every ten thousand cubic feet of space in the room.

To protect myself while looking after these cases, I used a pad of bichloride gauze as a germ sifter to breath through, and a long rubber coat to protect my clothing. After each visit I bathed my hands, face and hair in an antiseptic solution.

Vaccination should, of course, be practiced. The efficacy of vaccination was shown in its greatest glory during this epidemic. The disease would enter large families and limit itself to those members who had never been successfully vaccinated.

In conclusion, I will say that smallpox is a disease that can easily be prevented by vaccination. Can be managed successfully by quarantine—by preventing the entrance of infected material into a healthy community—the most dangerous material being bedding. Where the disease exists in a community, local quarantine of the infected premises is to be preferred to sending the patient to a pest house, this latter method being almost equivalent to homicide. The majority of patients in this Territory that were sent to pest houses died, while those that were left in their own homes recovered. In the treatment of the patient himself, strict attention to his personal hygiene and the sanitary arrangement of the sick chamber, together with the liberal use of antiseptics, stimulants and nourishing food, will rob the disease of much of its loathsomeness and lessen the death rate very materially.

